# The GCP16-zDHHC9 S-acyltransferase complex: Reciprocal effects on protein S-acylation and stability

**DOI:** 10.1016/j.jbc.2026.113349

**Published:** 2026-07-21

**Authors:** Despoina Allagioti, Andrew J. Thompson, Mohammad A. Al-Mhadeen, Kimon Lemonidis, Christine Salaun, Nicholas C.O. Tomkinson, Rami N. Hannoush, Shernaz X. Bamji, Luke H. Chamberlain

**Affiliations:** 1 Strathclyde Institute of Pharmacy and Biomedical Sciences, University of Strathclyde, Glasgow, United Kingdom; 2 Department of Cellular and Physiological Sciences, Life Sciences Institute and Djavad Mowafaghian Centre for Brain Health, University of British Columbia, Vancouver, British Columbia, Canada; 3 Faculty of Pharmacy and Medical Sciences, University of Petra, Amman, Jordan; 4 School of Molecular Biosciences, College of Medical Veterinary and Life Sciences, University of Glasgow, Glasgow, United Kingdom; 5 Department of Pure and Applied Chemistry, University of Strathclyde, Glasgow, United Kingdom; 6 Department of Early Discovery Biochemistry, Genentech, South San Francisco, California, USA

**Keywords:** accessory protein, palmitoylation, protein stability, S-acylation, zDHHC enzymes

## Abstract

S-Acylation is catalyzed by a family of twenty-three zinc-dependent zDHHC enzymes. A subset of these enzymes is regulated by accessory proteins, such as GCP16, which associates with the Golgi-localized acyltransferase zDHHC9 to form a functional complex. However, the specific bidirectional effects of the zDHHC9-GCP16 interaction in mammalian cells are not well defined. The S-acylation of both zDHHC9 and GCP16 is important for the activity of the acyltransferase complex. Here, we show that GCP16 enhances both the S-acylation and stability of zDHHC9, but does not affect another Golgi enzyme, zDHHC3. In addition, zDHHC9 mediates a reciprocal enhancement of the S-acylation and stability of GCP16. Similar effects on GCP16 are seen with zDHHC3, suggesting that increased S-acylation stabilizes GCP16. The S-acylated cysteines in GCP16 are present in a mostly hydrophobic region containing the α2′ and α3′ helices. This protein region appears to be linked to rapid proteasomal degradation and may become stabilized by S-acylation. GCP16 proteins with amino acid substitutions in the zDHHC9 binding interfaces confirmed the importance of complex formation for the bidirectional effects on S-acylation and stability. Furthermore, GCP16 with substitutions in the zDHHC9 binding interface did not support dendritic growth in hippocampal neurons, highlighting the functional importance of the intact complex. Overall, this study provides new insights into the bidirectional regulation of zDHHC9-GCP16 and reveals a role for S-acylation in protecting GCP16 from premature degradation. These effects may ensure that stable, functional and fully S-acylated zDHHC9-GCP16 complexes predominate at the Golgi over less stable noncomplexed zDHHC9 and GCP16.

S-Acylation is a reversible post-translational modification in which acyl chains from fatty acyl-CoA donors are attached to cysteine residues (1, 2). This post-translational modification is present throughout the eukaryotic kingdom, from yeast to humans, on thousands of transmembrane and soluble proteins (3) and affects membrane association, protein interactions, and protein stability (1, 2). This impacts many important cellular pathways, including synaptic signaling and plasticity (4, 5, 6).

S-Acylation is regulated by the opposing actions of S-acyltransferases and deacylases, which add and remove acyl chains, respectively (7, 8). The S-acyltransferases are a family of 23 polytopic membrane proteins defined by the presence of a 51-amino-acid cysteine-rich domain containing an aspartate-histidine-histidine-cysteine (DHHC) motif, and are called “zDHHC” enzymes (9, 10). The DHHC domain is the catalytic center of the enzymes and is stabilized by the binding of two zinc ions (10). The reaction mechanism involves the covalent attachment of an acyl chain onto the DHHC cysteine of the enzyme (referred to as autoacylation), followed by transfer and attachment to the target cysteine of a substrate protein (11, 12). Because autoacylation is a key intermediate in the reaction cycle, enzyme S-acylation status is often used as an indirect measurement of activity.

The majority of zDHHC enzymes are believed to be spontaneously active, but some isoforms require accessory proteins for full activity (13). This was first demonstrated in the yeast *Saccharomyces cerevisiae*, where the zDHHC enzyme ERF2 was shown to depend on the accessory protein ERF4 for activity (14, 15). The purified complex of both proteins was required for S-acylation of yeast Ras, and ERF4 was shown to stabilize the autoacylated state of ERF2 (16). Furthermore, levels of ERF2 were reduced in ERF4 mutant strains and showed enhanced ubiquitination, suggesting that the enzyme is also stabilized by ERF4 (16). The mammalian orthologues of ERF2 and ERF4 were subsequently identified as zDHHC9 and GCP16, respectively (17). Both of these proteins localize to the Golgi complex when expressed in mammalian cell lines (17). Interestingly, mutations in *ZDHHC9* have been shown to cause intellectual disability and childhood epilepsy (18, 19), which may be linked to the role of this enzyme in promoting dendrite growth (*via* S-acylation of Ras) and formation of inhibitory synapses (*via* S-acylation of TC10) (20).

A recent cryo-EM study reported the structure of the GCP16-zDHHC9 complex (21). This study identified four main interfaces involved in the interaction. For clarity and consistency with the results presented in that study, we also refer to these as interfaces 1, 2, 3, and 4. The amino acids involved in each of these interface interactions are highlighted in Supporting Information Fig. S1. Interface 1 involves interactions between the second and third transmembrane helices of zDHHC9, with Arginine-85 in the second TMD donating a hydrogen bond to the main chain of Tyrosine-76 in the membrane-embedded α3′ helix of GCP16, and Tyrosine-183 in the third transmembrane domain also interacting with Tyrosine-76 *via* π–π stacking. Interface 2 involves interactions between a type II polyproline (PPII) helix in the C-terminal region of zDHHC9 with α-helices in GCP16. Three prolines in this region of zDHHC9 are important, with Proline-290 and Proline-293 docking into two pockets formed by α1′ and α4′ helices in GCP16, and Proline-292 forming a CH-π hydrogen bond with Tyrosine-86 in GCP16. Interface 3 involves zinc finger motifs in the DHHC cysteine-rich domain of zDHHC9, where Phenylalanine-129 and Proline-150 form π–π stacking and CH-π interactions with Tyrosine-18 in GCP16. In addition, Glutamate-163 in zDHHC9 forms charge-charge interactions with Arginine-16 in GCP16. Arginine-16 and Tyrosine-18 occur in a loop region that follows the β1′ strand of GCP16. Finally, interface 4 involves electrostatic interactions between D100 and E101 of zDHHC9 with K11, R118 and R121 of GCP16, hydrophobic interactions between E101 and F104 of zDHHC9 with F13 of GCP16 and a salt bridge between E101 of zDHHC9 and R121 of GCP16. Interfaces 3 and 4 were suggested to contribute to stabilizing the zinc finger motifs in zDHHC9.

S-Acylation of both zDHHC9 and GCP16 at multiple sites is important for the activity of the acyltransferase complex. GCP16 is synthesized as a soluble protein, and its membrane association was reported to be mediated by S-acylation because alanine substitution of the S-acylated residues Cysteine-69 and Cysteine-72 reduced Golgi association (22). In the GCP16-zDHHC9 complex, Cysteine-69 and Cysteine-72 are located at the membrane interface in a cysteine cluster that also includes Cysteine-288 from zDHHC9. Alanine substitution of these cysteines in GCP16 and zDHHC9 leads to a loss of S-acyltransferase activity against H-Ras (21). In addition, zDHHC9 is also S-acylated at Cysteine-24 and Cysteine-25, and these residues are also important for enzyme activity. In this case, S-acylation of these cysteines anchors this region of zDHHC9 at the lipid bilayer and stabilizes its conformation (21). These data show that S-acylation of zDHHC9 at multiple cysteines (including the active site cysteine in the DHHC domain) is important for its S-acyltransferase activity.

To date, the effects of the GCP16-zDHHC9 interaction on both proteins in mammalian cells have not been clearly defined. Initial work on the recombinant purified GCP16-zDHHC9 complex reported that there was proteolysis of zDHHC9 in the absence of GCP16, and that GCP16 is essential for the enzymatic activity of zDHHC9 against Ras (17). Like ERF2-ERF4, purified zDHHC9 had a faster rate of active site acyl chain hydrolysis when GCP16 was absent (23). In addition, a further study showed that zDHHC9 levels were decreased and that the protein aggregated in the absence of GCP16, as shown by fluorescence-detection size-exclusion chromatography, and that there was an increase in the monodispersity and expression of zDHHC9 when GCP16 was coexpressed in Human Embryonic Kidney (HEK) 293 cells (24). Sequence similarity analyses have highlighted that zDHHC5, zDHHC8, zDHHC14, zDHHC18 and zDHHC19 are closely related to zDHHC9 (25, 26). Coimmunoprecipitation experiments revealed that endogenous GCP16 formed complexes with all of these enzymes except zDHHC19, and structural analysis of the zDHHC5-GCP16 interaction showed that interaction interfaces were similar to the zDHHC9-GCP16 complex (25, 27). In addition, GCP16 coexpression also reduced the aggregation of zDHHC5, zDHHC8, zDHHC14 and zDHHC18 (24), and enhanced the activity of zDHHC14 and zDHHC18 (21, 24). Collectively, these data show that GCP16 functionally regulates several zDHHC enzymes.

Overall, results to-date suggest that GCP16 forms complexes with zDHHC9 and related enzymes, stabilizing them and their S-acylated state, but this has not been demonstrated in intact mammalian cells. Furthermore, the reciprocal effects of zDHHC9 (and the related zDHHC enzymes) on GCP16 have not been reported, and this is especially relevant given that the accessory protein is S-acylated and that this modification is linked to the activity of the zDHHC9-GCP16 complex.

Given the importance of GCP16 for zDHHC9 activity, we have sought to provide a clearer understanding of the bidirectional regulation of the S-acylation and stability of these proteins in cells. We have also investigated how the different binding interfaces contribute to this bidirectional regulation and examined the importance of correct zDHHC9-GCP16 complex engagement for dendrite growth in hippocampal neurons.

## Results

### Bidirectional regulation of GCP16 and zDHHC9 S-acylation

S-Acylation of the active site cysteine of all zDHHC enzymes is essential for their catalytic activity. In addition, zDHHC9 is modified at several other cysteines, which are all important for the activity of the zDHHC9-GCP16 complex (21, 24). Furthermore, GCP16 is synthesized as a soluble protein, and its Golgi localization is disrupted by alanine substitution of the S-acylated cysteines at amino acid positions 69 and 72 (22). While S-acylation plays an important role in the function of both proteins, it has yet to be determined whether zDHHC9 mediates the S-acylation of GCP16 or whether GCP16 modulates the S-acylation status of zDHHC9 in mammalian cells.

**Figure 1:**
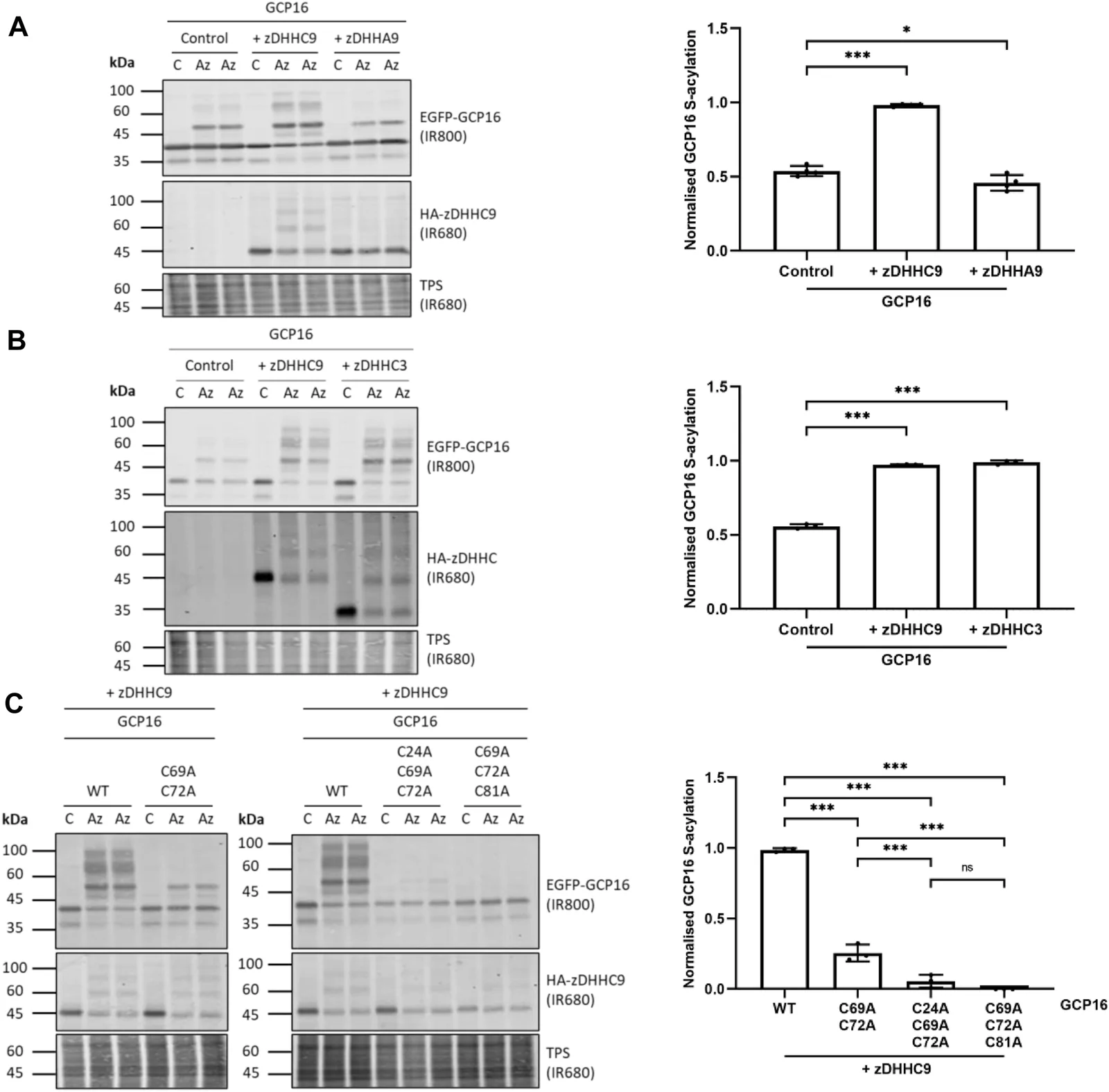
**Analysis of GCP16 S-acylation.** HEK293T cells were cotransfected with plasmids encoding (*A*) EGFP-GCP16 together with PEF-BOS-HA empty plasmid as control, HA-zDHHC9, or HA-zDHHA9, or (*B*) EGFP-GCP16 together with PEF-BOS-HA empty plasmid as control, HA-zDHHC9, or HA-zDHHC3, or (*C*) with HA-zDHHC9, together with EGFP-GCP16 WT, GCP16 (C69A/C72A), GCP16 (C24A/C69A/C72A), or GCP16 (C69A/C72A/C81A). Cells were labelled with palmitic acid azide (Az-C16:0) (Az) or palmitic acid (C16:0) as a control (C) for 4 h and were then lysed and clicked using Alkyne-mPEG. Samples were resolved by SDS-PAGE, transferred to nitrocellulose, and analyzed by a TPS followed by immunoblotting with GFP and HA antibodies. S-acylation is indicated by band shifts in Az samples. The position of molecular weight markers (kDa) is shown on the left of all immunoblots. The unlabeled zDHHC3 and zDHHC9 (control samples) migrate at approximately 35 kDa and 45 kDa, respectively. Quantified data show normalized protein S-acylation (mean ± SD). S-acylation (%) was calculated as a proportion of acylated and non-acylated band intensities. Data were normalized to the highest value in each experiment (set to 1), and then duplicates were averaged. Statistical significance was determined using one-way ANOVA, followed by a Dunnett’s multiple comparisons test (*A* and *B*), or a Tukey’s multiple comparisons test (*C*). ∗*p* < 0.05 and ∗∗∗*p* < 0.001, while ns denotes non-significance, where *p* > 0.05 (*p* values were <0.0001 (zDHHC9) and 0.0249 (zDHHA9) in panel (*A*), <0.0001 in panel (*B*), and <0.0001 (GCP16 (C69A/C72A), GCP16 (C24A/C69A/C72A), and GCP16 (C69A/C72A/C81A)), 0.0009 (GCP16 (C69A/C72A) *versus* GCP16 (C24A/C69A/C72A)), 0.0002 (GCP16 (C69A/C72A) *versus* GCP16 (C69A/C72A/C81A)), and 0.3610 (GCP16 (C24A/C69A/C72A) *versus* GCP16 (C69A/C72A/C81A)) in panel (*C*)). n = 4, from four independent experiments (*A*) or n = 3 from three independent experiments (*B* and *C*). Az, azide; TPS, total protein stain.

We first examined whether zDHHC9 can S-acylate GCP16. To do this, EGFP-GCP16 was coexpressed in HEK293T cells with either a control plasmid, HA-zDHHC9 WT or a catalytically inactive version of this enzyme (zDHHA9). The transfected cells were incubated with palmitic acidazide (Az) for 4 h (or palmitic acid as a control), lysed, and reacted with alkyne-tagged 5 kDa mPEG. The reaction of the Az (on the fatty acid) and alkyne (on the mPEG) groups leads to a band shift on SDS-PAGE gels for every attached palmitic acid azide molecule (28). Figure 1*A* shows that WT zDHHC9 induced a robust increase in the S-acylation of GCP16, which is indicated by the higher molecular weight bands that appear in the samples from cells incubated with palmitic acid azide, and a corresponding decrease in the amount of the non-acylated band in these samples (compare Az lanes with control (C) lanes). At least three band shifts were detected in samples from cells labelled with palmitic acid azide, suggesting that GCP16 is modified on at least three cysteines. In contrast, the catalytically inactive zDHHA9 construct did not enhance GCP16 S-acylation and instead caused a small but significant decrease in the S-acylation of the accessory protein (Fig. 1*A*). EGFP-GCP16 was also coexpressed with the broad specificity zDHHC3 enzyme, which, like zDHHC9, is localized at the Golgi (17, 29, 30, 31). Figure 1*B* shows that zDHHC3 produced a robust increase in GCP16 S-acylation. An increase in GCP16 S-acylation was also observed with zDHHC5 and zDHHC14 coexpression, and these enzymes have previously been reported to form functional complexes with GCP16 (Fig. S2) (21, 24, 25). In contrast, there was no effect of co-expression of zDHHC19 on GCP16 S-acylation (Fig. S2), consistent with the observation that these proteins do not coimmunoprecipitate in HEK293 cells (25).

GCP16 has four cysteine residues at amino acid positions 24, 69, 72, and 81. Cys-69 and Cys-72 were previously reported to be the main sites of S-acylation (22). To confirm that these sites are modified by zDHHC9, we coexpressed this enzyme with a GCP16 protein that had C69A and C72A amino acid substitutions. Compared to WT GCP16, there was a marked loss of S-acylation of the C69A/C72A protein, although a S-acylated band shift was still detected (Fig. 1*C*). Introduction of an additional alanine substitution at either Cys-24 or Cys-81 caused a complete loss of GCP16 S-acylation. Thus, zDHHC9 S-acylates Cys-69/Cys-72 in GCP16 but can also modify the other two cysteines in this protein.

As previously reported (22), we also found that combined alanine substitution of Cys-69 and Cys-72 was associated with reduced membrane attachment of GCP16, as determined using a detergent-based protocol for the separation of cytosolic and membrane proteins (Supporting Information Fig. S3). The effectiveness of the fractionation protocol is shown by the enrichment of GAPDH in the cytosol fraction and calnexin in the membrane fraction. Although coexpression with zDHHC9 enhanced GCP16 membrane binding, the fraction of the C69A/C72A protein that was membrane-associated was still significantly lower than WT GCP16.

**Figure 2:**
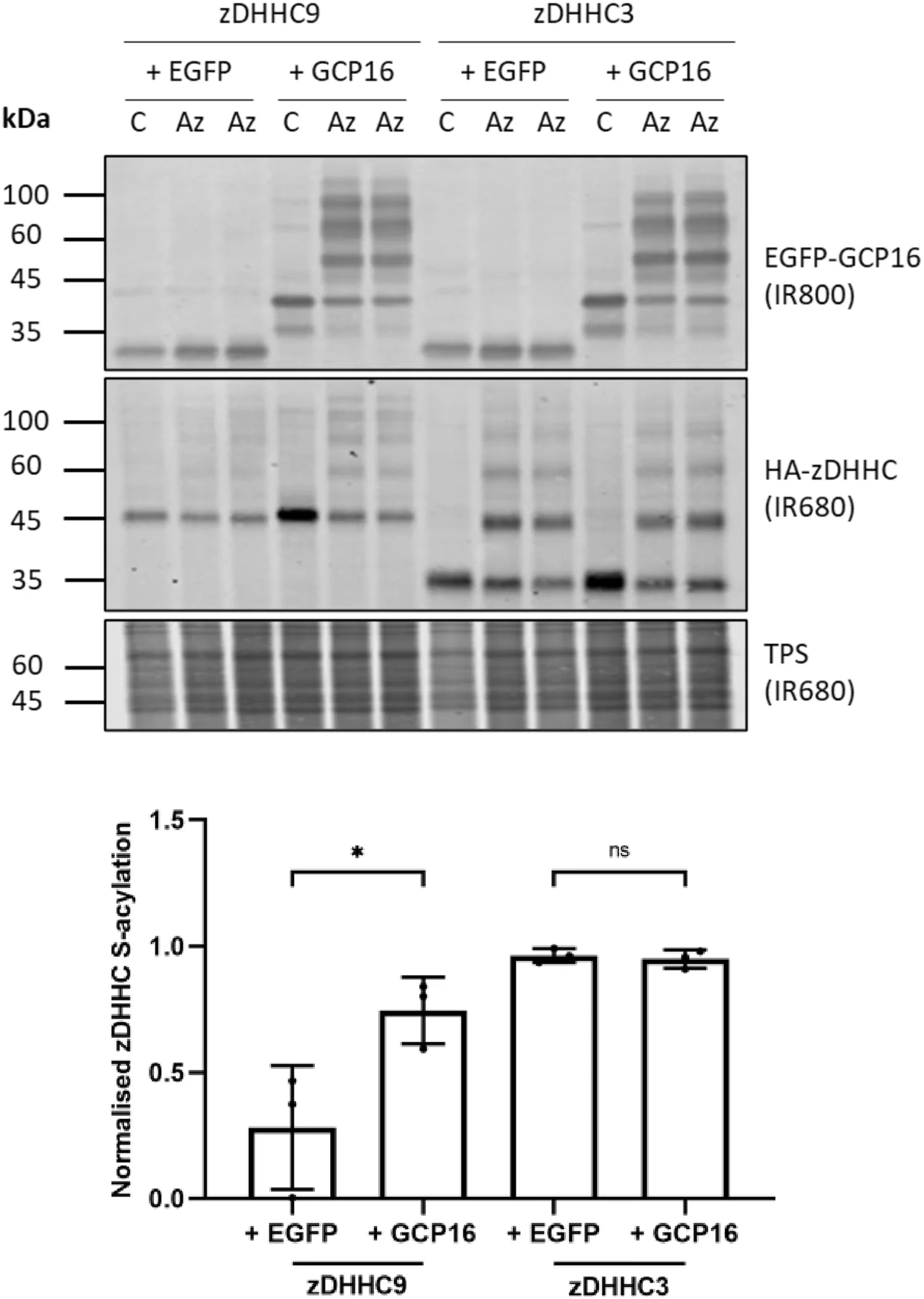
**Effects of GCP16 on the S-acylation of zDHHC9 and zDHHC3.** HEK293T cells were co-transfected with plasmids encoding HA-zDHHC9 or HA-zDHHC3 together with EGFP or EGFP-GCP16. Cells were labelled with palmitic acid azide (Az-C16:0) (Az) or palmitic acid (C16:0) as a control (C) for 4 h and were then lysed and clicked using Alkyne-mPEG. Samples were resolved by SDS-PAGE, transferred to nitrocellulose, and analyzed by a TPS followed by immunoblotting with GFP and HA antibodies. S-acylation is indicated by band shifts in Az samples. The position of molecular weight markers (kDa) is shown on the left of all immunoblots. Quantified data show normalized protein S-acylation (mean ± SD). S-acylation (%) was calculated as a proportion of acylated and non-acylated band intensities. Data were normalized to the highest value in each experiment (set to 1), then duplicates were averaged. Statistical significance was determined using an unpaired t-test. ∗*p* < 0.05, while ns denotes non-significance, where *p* > 0.05 (*p* values were 0.0448 (zDHHC9) and 0.6181 (zDHHC3)). n = 3, from three independent experiments. Az, azide; TPS, total protein stain.

Having examined the S-acylation of GCP16 by zDHHC enzymes, we next assessed the impact of GCP16 on the S-acylation of zDHHC9. S-acylation of zDHHC enzymes is correlated with their activity, as the “autoacylation” of the active site cysteine (*i.e.* the cysteine in the DHHC motif) is an essential intermediate required for substrate S-acylation. In addition to S-acylation of the active site, zDHHC enzymes are often modified on other cysteines, and indeed zDHHC9 is S-acylated on Cys-24/Cys-25 in the N-terminus and Cys-288 in the C-terminal tail, and these cysteine residues are also important for enzyme activity (21). We also examined the effect of GCP16 on zDHHC3, as there is no evidence of a functional interaction with this enzyme. zDHHC9 and zDHHC3 were coexpressed with either EGFP or EGFP-GCP16, and their S-acylation was assessed using click chemistry with 5 kDa mPEG. The results in Figure 2 show that GCP16 coexpression enhanced the S-acylation of zDHHC9. Furthermore, the profile of S-acylated bands (azide (Az) samples *versus* control (C) samples) confirmed that zDHHC9 S-acylation is stabilized at multiple sites. In contrast, GCP16 did not affect the S-acylation status of zDHHC3, which was robustly S-acylated at several sites, in both the absence and presence of the accessory protein.

Collectively, these analyses have highlighted bidirectional regulation of zDHHC9 and GCP16 S-acylation. In contrast, the regulatory relationship between zDHHC3 and GCP16 was unidirectional, with the enzyme enhancing S-acylation of GCP16 but not *vice versa*.

### Bidirectional regulation of GCP16 and zDHHC9 stability

The bidirectional regulation of zDHHC9 and GCP16 S-acylation prompted us to investigate if there was a similar bidirectional regulation of protein stability. In *S. cerevisiae*, ERF2 (zDHHC9 orthologue) is rapidly degraded in the absence of ERF4 (GPC16 orthologue) (16). In addition, zDHHC9 has been shown to have lower expression and to aggregate in cell lysates in the absence of GCP16, suggesting it is stabilized by its interaction with this accessory protein (24). However, the impact of GCP16 on zDHHC9 stability in cells has not been examined, and it remains unclear whether zDHHC9 reciprocally stabilizes GCP16.

**Figure 3:**
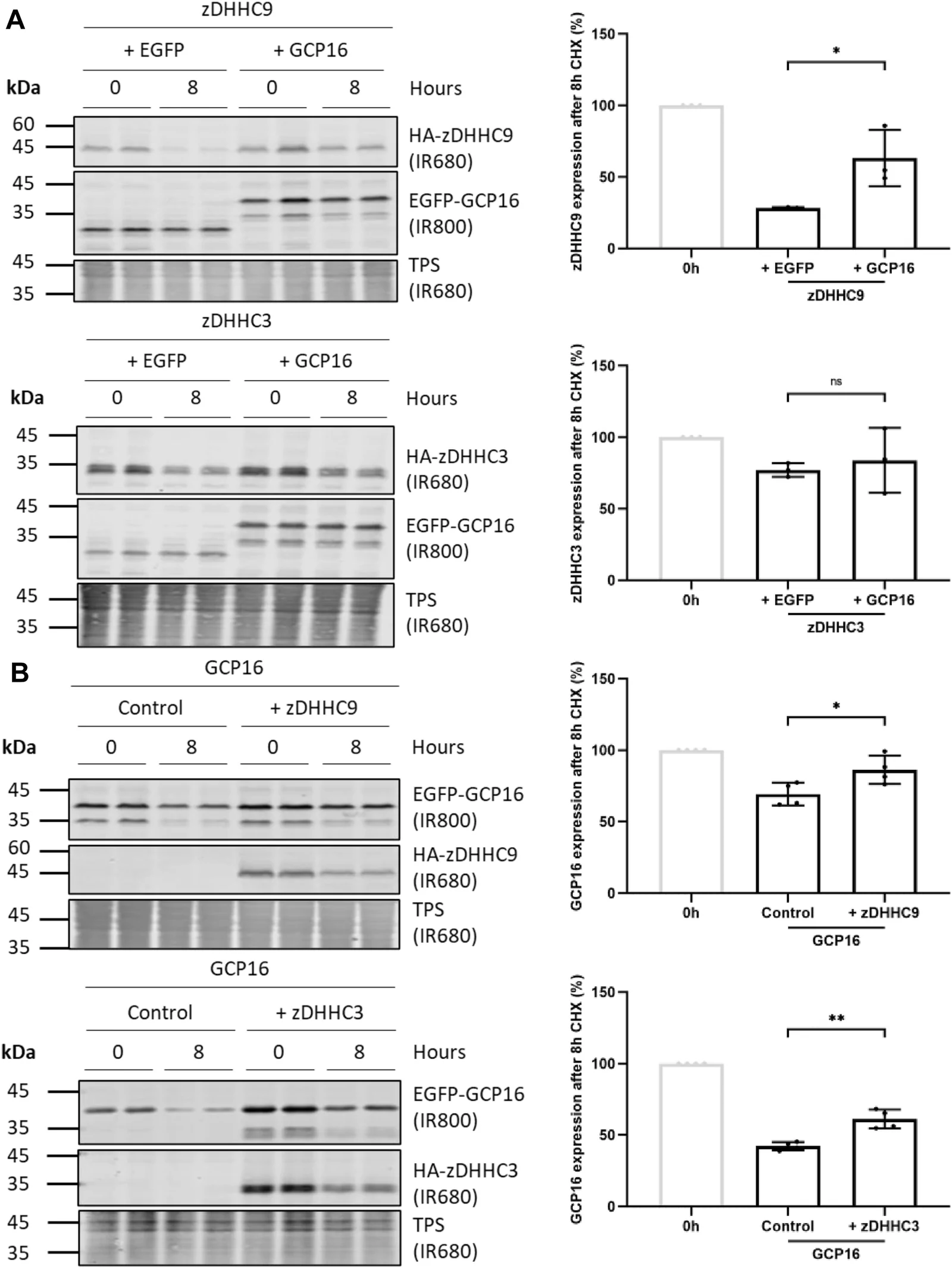
**Analysis of the effects of GCP16 on the stability of zDHHC9 and zDHHC3, and the effects of the enzymes on GCP16 stability.** HEK293T cells were cotransfected with (*A*) HA-tagged zDHHC9 or zDHHC3, together with either EGFP or EGFP-GCP16, or with (*B*) EGFP-GCP16 together with HA-zDHHC9 or HA-zDHHC3 (PEF-BOS-HA empty plasmid was used in control transfections). Cell lysates were collected at 0 h or after 8 h of incubation with 50 μg/ml CHX. Samples were resolved by SDS-PAGE, transferred to nitrocellulose, and analyzed by a TPS followed by immunoblotting with GFP and HA antibodies. The position of molecular weight markers (kDa) is shown on the left of all immunoblots. Percentage protein expression after 8 h of cycloheximide treatment is shown for each condition (mean ± SD). Signals were normalized to the TPS for each lane and then expressed relative to the corresponding 0 h value (set to 100%). Statistical significance was determined using unpaired t-tests. ∗*p* < 0.05 and ∗∗*p* < 0.01, while ns denotes non-significance, where *p* > 0.05 (*p* values in panel (*A*) were 0.0372 (zDHHC9) and 0.6373 (zDHHC3), while in panel (*B*) they were 0.0376 (zDHHC9) and 0.0020 (zDHHC3)). n = 3, from two independent experiments (*A*), or n = 4, from three (*top*) or two (*bottom*) independent experiments (*B*). CHX, cycloheximide; TPS, total protein stain.

We first examined the effect of GCP16 on the stability of zDHHC9 and included zDHHC3 for comparison. For this, the zDHHC enzymes were coexpressed with either EGFP or EGFP-GCP16, and cell lysates were collected before and following an 8-h treatment with the protein synthesis inhibitor cycloheximide. Figure 3*A* (*top panel*) shows that the reduction in zDHHC9 expression at 8 h was less when coexpressed with GCP16, suggesting that the accessory protein stabilizes this enzyme. In contrast, there was no change in the rate of degradation of zDHHC3 when coexpressed with GCP16 (Fig. 3*A*, *bottom panel*). We then investigated whether the zDHHC enzymes enhanced the stability of GCP16. In this case, both zDHHC9 and zDHHC3 coexpression reduced GCP16 degradation (Fig. 3*B*).

As with S-acylation, these findings highlight a bidirectional protein stability relationship between GCP16 and zDHHC9 and a unidirectional relationship between GCP16 and zDHHC3. The stabilizing effect of both zDHHC9 and zDHHC3 implies that S-acylation promotes the stability of GCP16. For zDHHC9, stabilization either comes directly from entry into a complex with GCP16 or indirectly through the increased S-acylation that complex formation invokes.

### The amino acid region 60 to 90 is linked to GCP16 degradation

**Figure 4:**
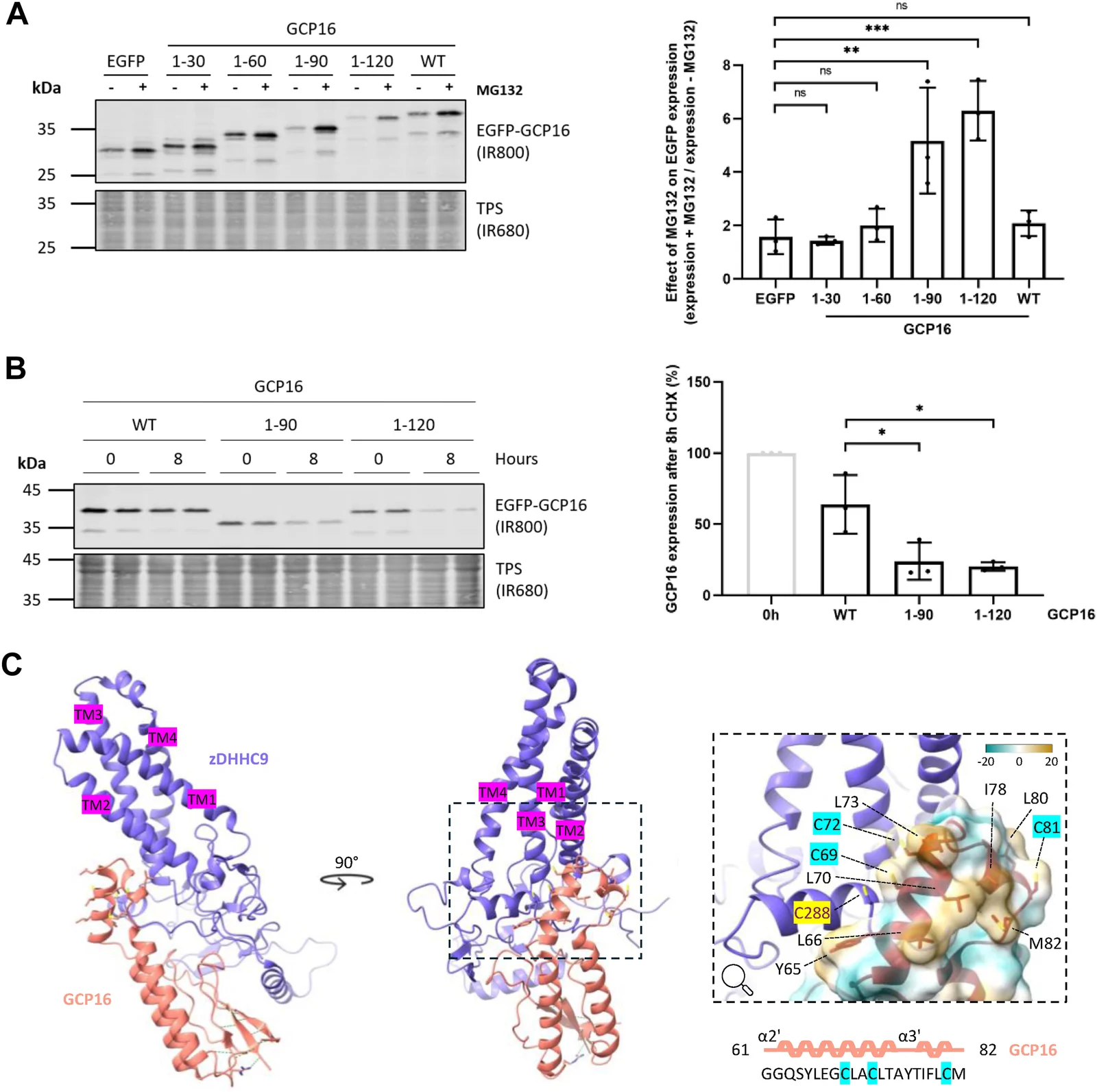
**The amino acid region 60 to 90 in GCP16 promotes proteasomal degradation.***A,* HEK293T cells were transfected with EGFP, EGFP-GCP16 1-30, 1-60, 1-90, 1-120, or GCP16 WT. Cells were incubated with either DMSO as a vehicle control (−) or with MG132 (10 μM) (+) for 16 h. Cells were lysed, samples resolved by SDS-PAGE and transferred to nitrocellulose, and analyzed by a TPS followed by immunoblotting with GFP antibody. Quantified data show protein expression after MG132 (mean ± SD), with signals normalized to TPS and expressed relative to matched DMSO controls. *B,* HEK293T cells were transfected with EGFP-GCP16 WT, 1-90, or 1-120. Cell lysates were collected at 0 h or after 8 h of incubation with 50 μg/ml CHX. Samples were resolved by SDS-PAGE, transferred to nitrocellulose, and analyzed by a TPS followed by immunoblotting with GFP antibody. Percentage protein expression after 8 h of CHX treatment is shown for each condition (mean ± SD). Signals were normalized to the TPS for each lane and then expressed relative to the corresponding 0 h value (set to 100%). Statistical significance was determined using one-way ANOVA, followed by a Dunnett’s multiple comparisons test. ∗*p* < 0.05, ∗∗*p* < 0.01 and ∗∗∗*p* < 0.001, while ns denotes nonsignificance, where *p* > 0.05 (in panel (*A*), *p* values were 0.9997 (GCP16 1–30), 0.9782 (GCP16 1–60), 0.0041 (GCP16 1–90), 0.0005 (GCP16 1–120), and 0.9581 (GCP16 WT), while in panel (*B*) they were 0.0248 (GCP16 1–90) and 0.0170 (GCP16 1–120)). n = 3, from two independent experiments (*A*), or from three independent experiments (*B*). The position of molecular weight markers (kDa) is shown on the left of all immunoblots. *C,* cartoon representation of the zDHHC9-GCP16 protein complex (PDB: 8HF3). zDHHC9 is shown in slate blue and GCP16 is shown in salmon (or *dark red* in close-up view). The hydrophobic residues of the α2′ and α3′ helices of GCP16 are depicted as sticks, while GCP16’s surface is colored by hydrophobicity. The residues making up the two helices are depicted in the schematic below, and cysteine residues are highlighted in turquoise. The TM domains of zDHHC9 are highlighted in *pink*, while Cysteine-288 which is in proximity to the α-helices of GCP16 is highlighted in *yellow*. CHX, cycloheximide; TM, transmembrane; TPS, total protein stain.

The data presented in the preceding section suggest that GCP16 is stabilized by S-acylation. We therefore investigated whether there is a specific region of GCP16 that drives degradation in the absence of S-acylation. For this, we fused increasing lengths of the N-terminal region of GCP16 to EGFP and monitored expression levels in the absence and presence of the proteasome inhibitor MG132. Interestingly, whereas amino acids 1-30 and 1-60 from GCP16 did not affect EGFP levels, addition of residues 1-90 and 1-120 substantially reduced the EGFP signal, and this was restored by MG132 (Fig. 4*A*), suggesting that the amino acid region 60 to 90 drives rapid proteasomal degradation. The quantified data in Figure 4*A* shows protein expression in the presence of MG132 relative to expression in the absence of MG132. Rapid proteasomal degradation of the 1-90 and 1-120 GCP16 mutants was also suggested by cycloheximide chase experiments (Fig. 4*B*). It is interesting that amino acids 60 to 90 contain the main S-acylated cysteines in GCP16 and include regions that are notably hydrophobic. Indeed, the cryo-EM structure of zDHHC9-GCP16 (PDB: 8HF3) reveals membrane interaction of the α2′ and α3′ helices of GCP16 (encompassing amino acid residues 61–82) and that they are adjacent to the TMDs of zDHHC9 (Fig. 4*C*).

Click chemistry assays showed that the rapidly degrading GCP16 1 -90 and 1-120 proteins were not S-acylated in either the absence or presence of zDHHC9 (Supporting Information Fig. S4). Collectively, these results suggest that the 60 to 90 region of GCP16, which includes the α2′ and α3′ helices and the main S-acylated cysteines, may drive degradation of the protein when present in a nonacylated state.

### Disrupting the binding interfaces of GCP16 and zDHHC9 perturbs S-acylation and stability of both proteins

Recent work by Yang *et al.* (2024) identified key residues involved in the zDHHC9-GCP16 interaction, which occurs at four main interfaces (referred to as interfaces 1–4; see Supporting Information Fig. S1). To examine how these interfaces contribute to the bidirectional regulatory effects on GCP16 and zDHHC9, we introduced alanine substitutions at the key interacting residues. Note that although E124 in GCP16 is not directly involved in zDHHC9 binding, it does stabilize R118 in the binding interface, and so we included an alanine substitution at this position to further disrupt interface 4 (21). We initially examined the effects of combined disruption of all four interfaces in either zDHHC9 or GCP16. This was associated with a loss of the bidirectional effects on both S-acylation (Supporting Information Figs. S5 and S6) and stability (Supporting Information Figs. S7 and S8).

**Figure 5:**
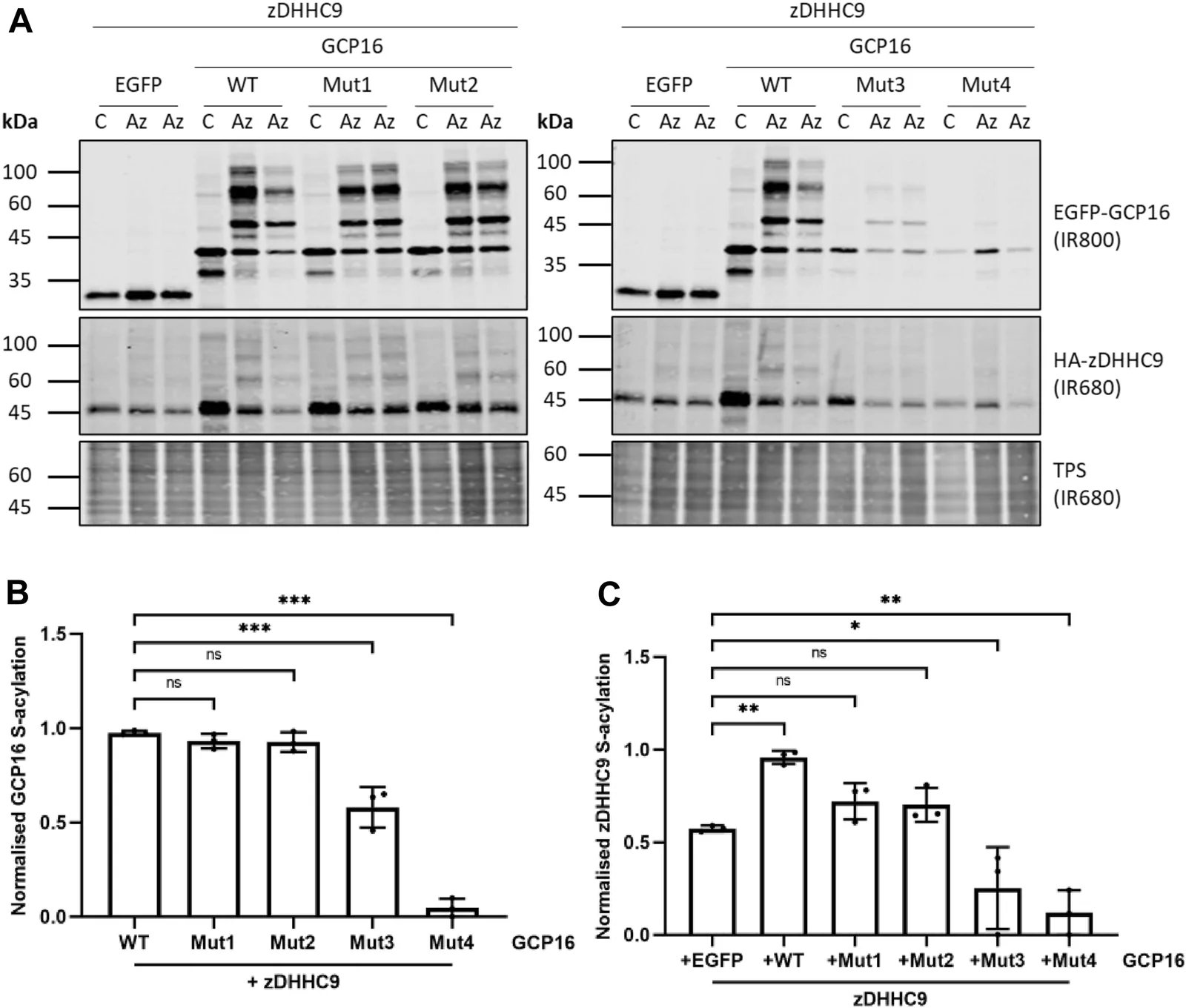
**Effects of amino acid substitutions in the binding interfaces of GCP16 on the S-acylation of GCP16 and zDHHC9.***A,* HEK293T cells were cotransfected with plasmids encoding HA-zDHHC9, together with either EGFP, EGFP-GCP16 WT, or the EGFP-tagged GCP16 binding interface mutants 1, 2, 3, or 4. Cells were labelled with palmitic acid azide (Az-C16:0) (Az) or palmitic acid (C16:0) as a control (C) for 4 h and were then lysed and clicked using Alkyne-mPEG. Samples were resolved by SDS-PAGE, transferred to nitrocellulose, and analyzed by a TPS followed by immunoblotting with GFP and HA antibodies. S-acylation is indicated by band shifts in Az samples. The position of molecular weight markers (kDa) is shown on the left of all immunoblots. *B* and *C,* quantified data show normalized protein S-acylation (mean ± SD). S-acylation (%) was calculated as a proportion of acylated and nonacylated band intensities. Data were normalized to the highest value in each experiment (set to 1), then duplicates were averaged. Statistical significance was determined using one-way ANOVA, followed by a Dunnett’s multiple comparisons test. ∗*p* < 0.05, ∗∗*p* < 0.01 and ∗∗∗*p* < 0.001, while ns denotes nonsignificance, where *p* > 0.05 (in panel (*B*), *p* values were 0.7936 (GCP16 Mut1), 0.7448 (GCP16 Mut2) and <0.0001 (GCP16 Mut3 and GCP16 Mut4), while in panel (*C*) they were 0.0072 (GCP16 WT), 0.4392 (GCP16 Mut1), 0.5630 (GCP16 Mut2), 0.0236 (GCP16 Mut3), and 0.0021 (GCP16 Mut4)). n = 3, from three independent experiments. Az, azide; TPS, total protein stain.

To look more closely at the importance of each binding interface in GCP16, we examined GCP16 proteins with alanine substitutions at individual interfaces (Supporting Information Fig. S1*A*). Figure 5 shows that the S-acylation of GCP16 when coexpressed with zDHHC9 was significantly reduced when binding interfaces 3 and 4 were disrupted, whereas amino acid substitutions in interfaces 1 and 2 did not affect GCP16 S-acylation. In contrast, amino acid substitutions introduced into any of the four binding interfaces disrupted the ability of GCP16 to enhance the S-acylation of zDHHC9 above control levels (Fig. 5*C*). This suggests that stabilization of zDHHC9 S-acylation status is very sensitive to perturbations in GCP16 interaction, whereas S-acylation of GCP16 by this enzyme can tolerate minor disruptions to their interaction.

**Figure 6:**
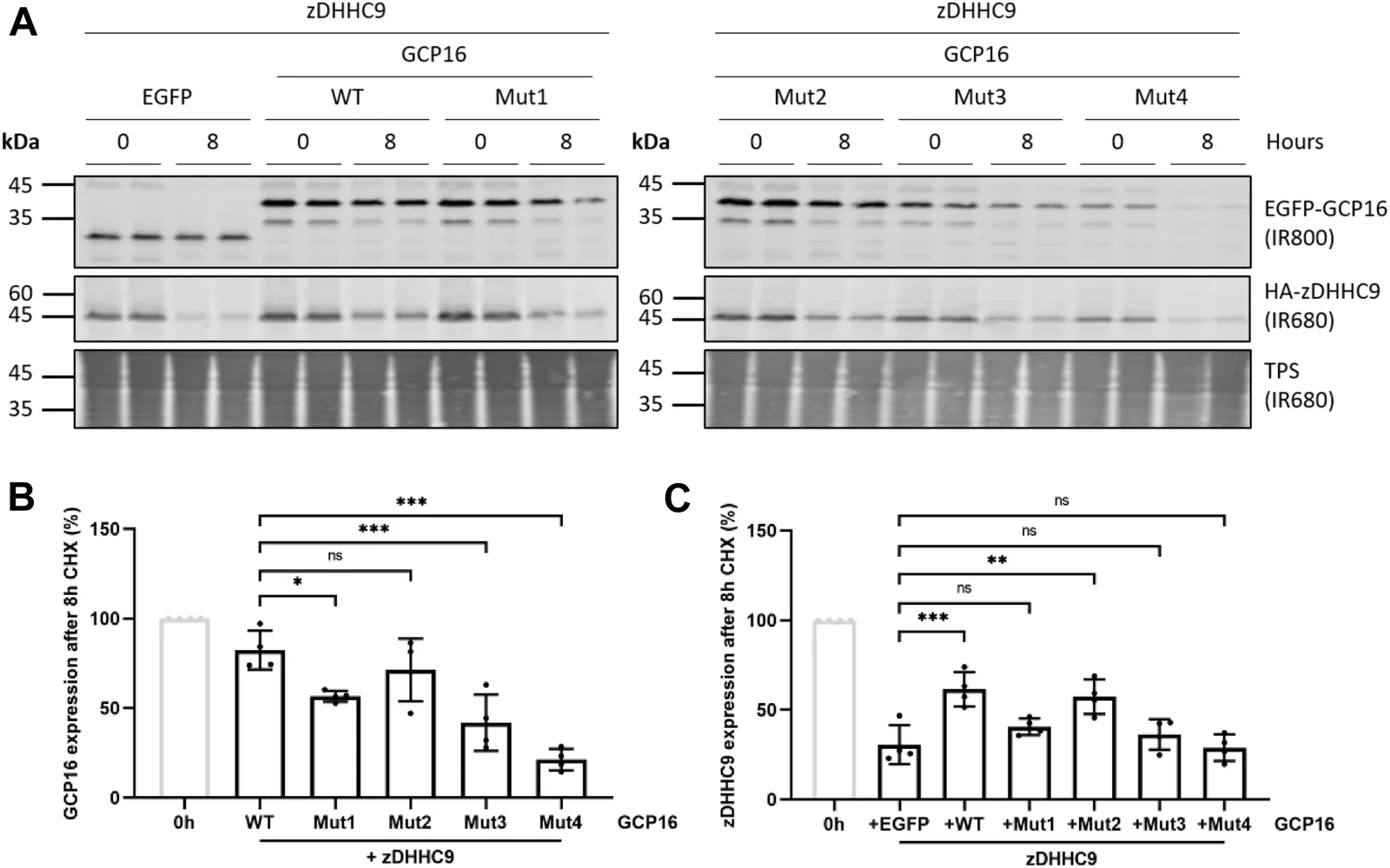
**Effects of amino acid substitutions in the binding interfaces of GCP16 on the stability of GCP16 and zDHHC9.***A,* HEK293T cells were cotransfected with plasmids encoding HA-zDHHC9, together with either EGFP, EGFP-GCP16 WT, or the EGFP-tagged GCP16 binding interface mutants 1, 2, 3, or 4. Cell lysates were collected at 0 h or after 8 h of incubation with 50 μg/ml CHX. Samples were resolved by SDS-PAGE, transferred to nitrocellulose, and analyzed by a TPS followed by immunoblotting with GFP and HA antibodies. The position of molecular weight markers (kDa) is shown on the left of all immunoblots. *B* and *C,* percentage protein expression after 8 h of CHX treatment is shown for each condition (mean ± SD). Signals were normalized to the TPS for each lane and then expressed relative to the corresponding 0 h value (set to 100%). Statistical significance was determined using one-way ANOVA, followed by a Dunnett’s multiple comparisons test. ∗*p* < 0.05, ∗∗*p* < 0.01 and ∗∗∗*p* < 0.001, while ns denotes non-significance, where *p* > 0.05 (*p* values in panel (*B*) were 0.0270 (GCP16 Mut1), 0.5122 (GCP16 Mut2), 0.0009 (GCP16 Mut3), and <0.0001 (GCP16 Mut4), while in panel (*C*) they were 0.0004 (GCP16 WT), 0.3670 (GCP16 Mut1), 0.0017 (GCP16 Mut2), 0.8091 (GCP16 Mut3), and 0.9984 (GCP16 Mut4)). n = 4, from four independent experiments. CHX, cycloheximide; TPS, total protein stain.

When the interface mutants of GCP16 were examined in cycloheximide chase experiments, it was seen that all mutants, except mutant 2, had a faster turnover rate than WT GCP16 when expressed together with zDHHC9 (Fig. 6*B*). The data in Figure 6*C* further show that the interface mutations that perturb GCP16 stability also disrupt the ability of GCP16 to stabilize zDHHC9.

### MG132 treatment suggests that GCP16 interface mutant 4 is rapidly degraded by the proteasome

**Figure 7:**
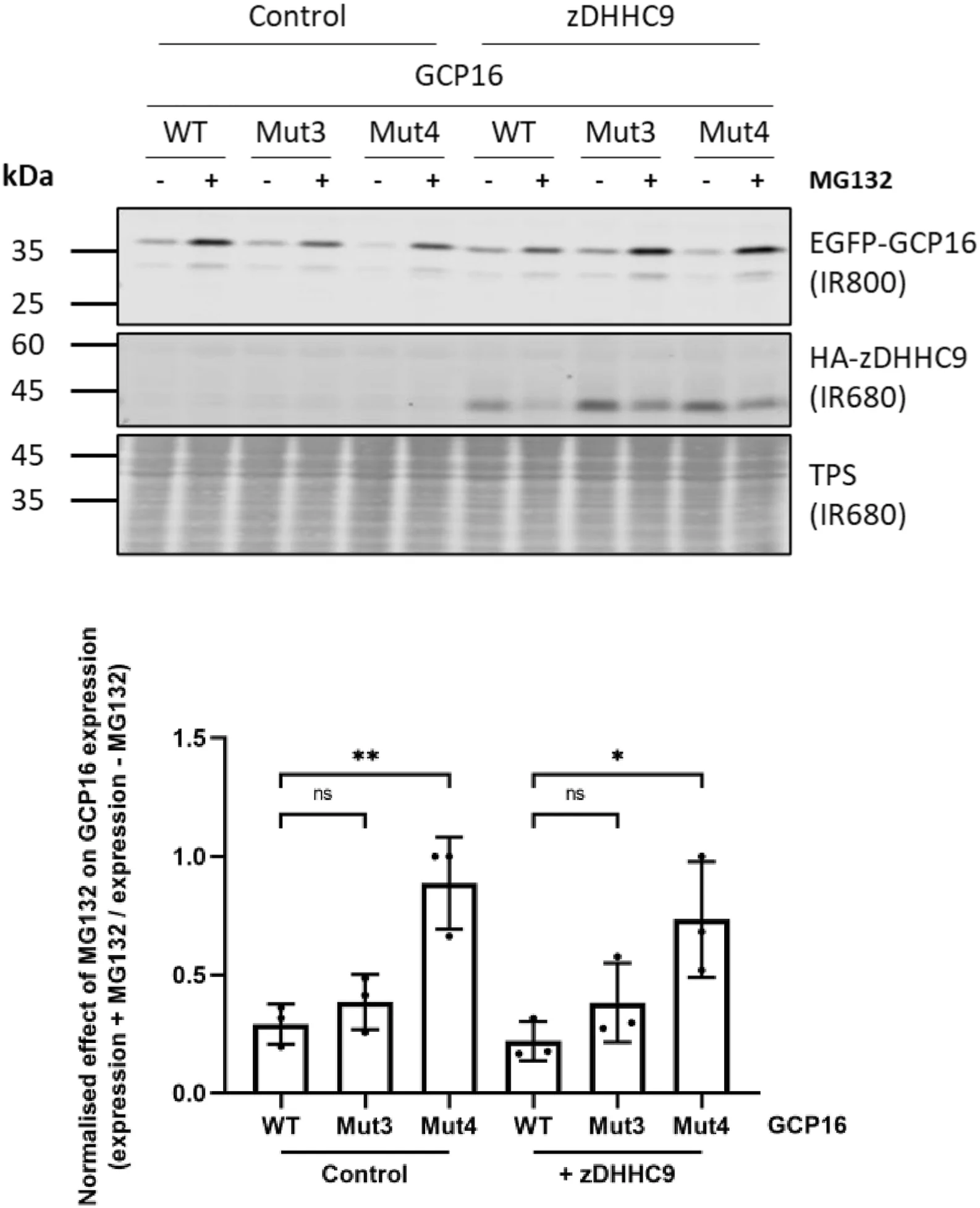
**Effect of MG132 proteasomal inhibition on GCP16 binding interface mutants.** HEK293T cells were transfected with HA-zDHHC9 or PEF-BOS-HA empty plasmid (control), together with EGFP, EGFP-GCP16 WT, or EGFP-tagged GCP16 binding interface mutants 3 or 4. Cells were incubated with either DMSO as a vehicle control (-) or with MG132 (10 μM) (+) for 16 h. Cells were lysed and samples were resolved by SDS-PAGE, transferred to nitrocellulose, and analyzed by a TPS followed by immunoblotting with GFP and HA antibodies. The position of molecular weight markers (kDa) is shown on the left of all immunoblots. Quantified data show normalized protein expression after MG132 (mean ± SD), with signals normalized to TPS and expressed relative to matched DMSO controls. Data were normalized to the highest value in each experiment (set to 1). Statistical significance was determined using one-way ANOVA, followed by a Dunnett’s multiple comparisons test. ∗*p* < 0.05 and ∗∗*p* < 0.01, while ns denotes nonsignificance, where *p* > 0.05 (*p* values in the control analysis were 0.6432 (GCP16 Mut3) and 0.0036 (GCP16 Mut4), while *p* values in the analysis with zDHHC9 were 0.4699 (GCP16 Mut3) and 0.0215 (GCP16 Mut4)). n = 3, from three independent experiments. TPS, total protein stain.

The previous results showed that GCP16 interface mutants 1, 3, and 4 had a decreased half-life when coexpressed with zDHHC9. To determine if this is linked to enhanced proteasomal degradation, cells were transfected with WT EGFP-GCP16 or interface mutants 3 or 4, with and without zDHHC9. The transfected cells were then incubated in the absence or presence of MG132. As shown in Figure 7, MG132 promoted a significant increase in expression of interface mutant 4, both with and without zDHHC9 coexpression, compared with its effects on WT GCP16. As expected, MG132 did not enhance expression of the multi-transmembrane zDHHC9 protein (Fig. 7). These observations imply that the reduced stability of interface mutant 4 is linked to its rapid proteasomal degradation.

### GCP16 interface mutant 3 fails to support dendrite growth in hippocampal neurons

**Figure 8:**
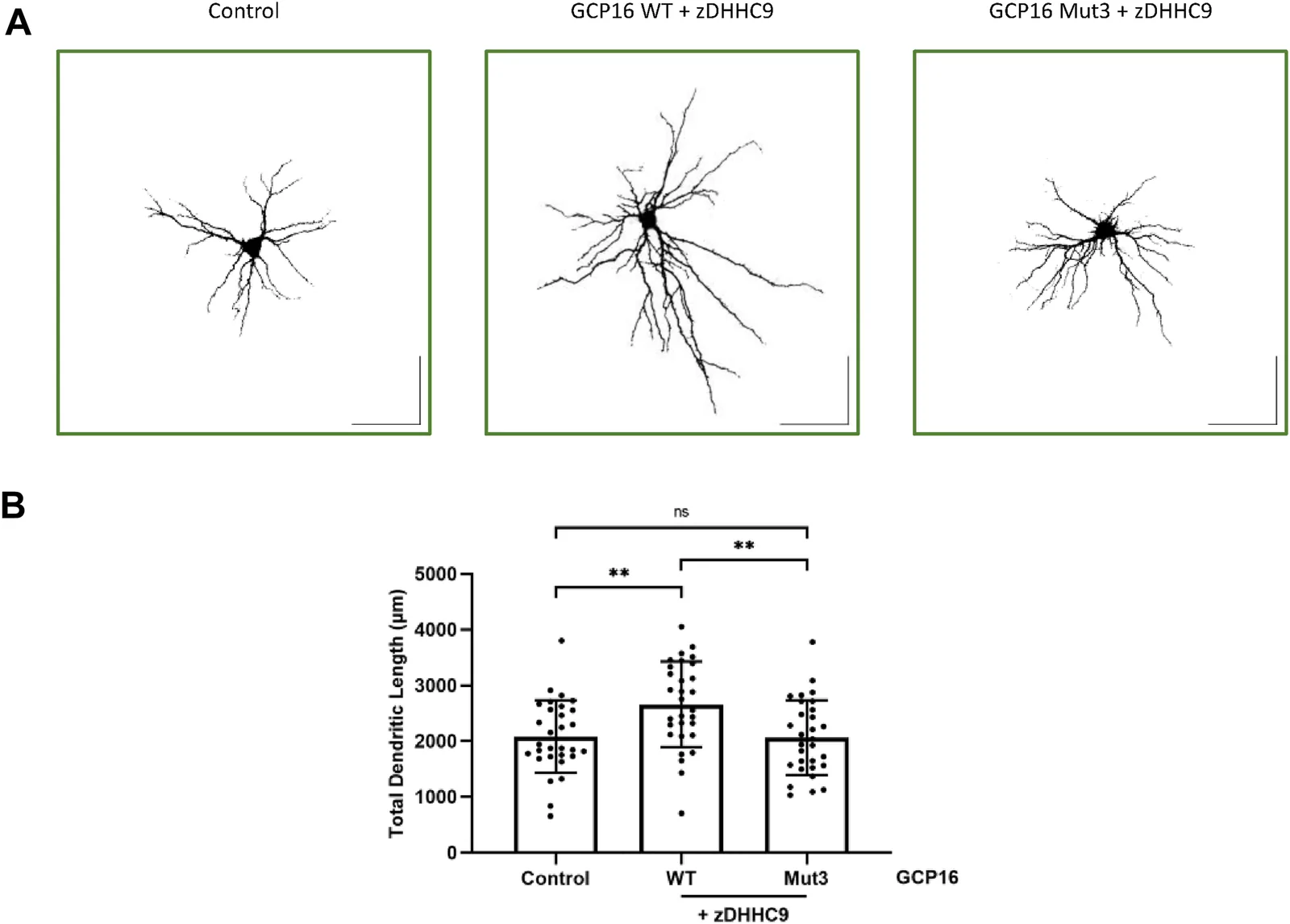
**GCP16 interface mutant 3 fails to support dendrite growth in rat hippocampal neurons.** Cultured rat hippocampal neurons were transfected at DIV 9 with HA-zDHHC9, FLAG-GCP16 WT or interface mutant 3, and Camk2a-EGFP plasmid constructs. Cells were fixed at DIV 14, permeabilized, and labelled with appropriate primary and secondary antibodies, and with DAPI. Cells were imaged on an Evident IX83 inverted microscope equipped with a Hamamatsu Orca Flash sCMOS camera and an X-Cite white light LED. *A,* representative binarized mask images (imaged at 20X magnification) of the EGFP channel of transfected rat hippocampal neurons, which was used to measure total dendritic length using the SNT plugin on Image J software. The scale bar represents = 100 μm. *B,* quantified data shows the mean total dendritic length (μm) (± SD) measured in SNT after manually tracing dendritic arbors using the mask images. Statistical significance was determined using one-way ANOVA followed by a Tukey’s multiple comparisons test. ∗∗*p* < 0.01, while ns denotes non-significance, where *p* > 0.05 (*p* values in panel (*B*) were 0.0055 (GCP16 WT), 0.9931 (GCP16 Mut3), and 0.0039 (GCP16 WT *versus* GCP16 Mut3)). n = 30, from three separate cultures. SNT, SimpleNeuriteTracer.

The data presented thus far show that complex formation between GCP16 and zDHHC9 exerts bidirectional regulation on S-acylation and protein stability. To confirm that these effects are functionally important, we examined how binding interface disruption affects the ability of GCP16 to support dendrite growth in hippocampal neurons. It has previously been shown that co-expression of GCP16 and zDHHC9 (but not either protein alone) enhances dendrite growth in this system (20). Hence, we compared the effects of WT FLAG-GCP16 and binding interface mutant 3 in this assay. Mutant 3 was selected as it had clear effects on S-acylation and stability, but not as severe as those of mutant 4. To limit any impact of different expression levels on the results obtained, transfected neurons selected for analysis had similar fluorescence intensity levels of zDHHC9 (*p* = 0.65) and GCP16 (*p* = 0.33) between WT and mutant 3 cells. As shown in Figure 8, WT GCP16 co-expressed with zDHHC9 promoted a significant increase in dendrite growth. In contrast, interface mutant 3 did not affect dendrite length. This provides evidence that the bidirectional regulation of GCP16 and zDHHC9 is essential for their function in neurons.

## Discussion

The results reported in this study suggest that: (i) GCP16 regulates the S-acylation status and stability of zDHHC9 in cells but has no effect on another Golgi-localized enzyme, zDHHC3; (ii) GCP16 is S-acylated by the related enzymes zDHHC9, zDHHC5 and zDHHC14, and also by the broad specificity zDHHC3 enzyme; (iii) GCP16 half-life is increased by both zDHHC9 and zDHHC3, suggesting that S-acylation stabilizes the accessory protein; (iv) The 60 to 90 amino acid region of GCP16 containing the α2′ and α3′ helices is linked to rapid proteasomal degradation and may be stabilized by S-acylation; (v) Disruption of specific binding interfaces perturbs the stability and S-acylation of GCP16 and zDHHC9, and confirms the bidirectional regulatory effects of zDHHC9-GCP16 complex formation; and (vi) Binding interface disruption leads to loss of function of the zDHHC9-GCP16 complex in dendrite growth. Based on these data, we propose that newly-synthesized GCP16 may initially associate with membranes through the hydrophobicity of α2′ and α3′ helices. However, the protein is unstable in this non-acylated form. At the membrane, it connects with zDHHC9 through specific binding interactions, promoting the S-acylation and stabilization of both proteins. The enhanced stability of the S-acylated complex may ensure that this predominates over the less stable noncomplexed forms of each protein.

The observation that GCP16 stabilizes zDHHC9 S-acylation is consistent with previous studies reporting that the accessory protein enhances the autoacylation of the purified enzyme (17, 21, 23). Our study extends these observations by showing that GCP16 also regulates zDHHC9 S-acylation status in cells. Furthermore, as several band shifts were observed in click-PEG assays, it appears that zDHHC9 S-acylation increases at multiple sites and not only the DHHC cysteine. GCP16 may protect the S-acylation of these other sites directly, for example, the formation of a cysteine cluster of Cys-69 and Cys-72 from GCP16 and Cys-288 from zDHHC9 may protect this S-acylated residue on the enzyme. As alanine substitutions of Cys-24, Cys-25 and Cys-288 led to a loss of zDHHC9 activity (21), it is logical to assume that the S-acylation status of zDHHC9 observed in our experiments provides an accurate assessment of the effects of GCP16 on zDHHC9 activity. As GCP16 binds to a surface of zDHHC9 that is opposite the DHHC domain (21), effects of GCP16 on the active site cysteine might occur secondary to structural stabilization of the enzyme. However, as interface mutant 2 of GCP16 can efficiently stabilize zDHHC9 but does not significantly affect the S-acylation of the enzyme, this suggests that the effects of GCP16 on the wider S-acylation profile of zDHHC9 may be more complex. These effects of GCP16 on enzyme S-acylation were not seen with zDHHC3, despite this enzyme also S-acylating the accessory protein; indeed, it was notable that zDHHC3 was more robustly S-acylated than zDHHC9 (even in the presence of GCP16), consistent with its high intrinsic level of activity against a broad range of substrates (32).

The selective effect of GCP16 on zDHHC9 was also apparent when enzyme stability was assessed using cycloheximide chase experiments. GCP16 slowed the rate of zDHHC9 degradation but did not affect zDHHC3. These findings extend the work of Nguyen *et al.* (2023), who demonstrated that zDHHC9 is present in higher molecular weight complexes (aggregates) in cell lysates in the absence of GCP16. The cycloheximide chase experiments performed here showed that WT GCP16 slowed the degradation of zDHHC9 over an 8-h time window, and this was prevented by introducing amino acid substitutions into binding interfaces 1, 3, and 4. Although GCP16 proteins with amino acid substitutions in these binding interfaces were less stable than WT GCP16, the effects of the interface mutants on zDHHC9 can be rationalized by considering the regions of zDHHC9 that are involved in these interactions. Binding interface 3 involves interactions with the zinc fingers of zDHHC9, whereas binding interface 4 is predicted to indirectly stabilize the zinc finger motifs (21). In addition, Tyr-76 (interface 1) interacts with residues in the second and third transmembrane domains of zDHHC9, which flank the intracellular loop containing the catalytic domain and zinc fingers. It has been shown previously that the zinc fingers are important for the structural integrity of zDHHC enzymes (33), which can explain why disrupting interactions of GCP16 in and around these zinc binding sites can perturb zDHHC9 stability. In contrast, Tyr-86 (interface 2) interacts with the PPII helix in the C-terminus of zDHHC9, which, while still important for the overall structure of the complex, may be less important for enzyme stability. Another possible reason for the lack of effect of the interface 2 mutant on protein stability is that this interface involves both the interaction of Pro-292 in the PPII helix of zDHHC9 with Tyr-86 in GCP16 and interactions of Pro-290 and Pro-293 with two negatively charged pockets of GCP16. Therefore, future work might look to generate more disruptive amino acid substitutions, including in the two pockets of GCP16. Nevertheless, the Y86A substitution did affect the ability of GCP16 to enhance the S-acylation of zDHHC9, showing that this single change is sufficient to disrupt the normal functional effects of protein complex formation.

Coexpression of GCP16 with zDHHC9, zDHHC5 or zDHHC14, which have been previously found to form a complex with GCP16 (21, 24, 25), led to an increase in the S-acylation of the accessory protein. In contrast, zDHHC19, which does not coimmunoprecipitate with GCP16 (25), had no effect on GCP16 S-acylation. This suggests that stable complex formation with GCP16 is a prerequisite for efficient S-acylation of the accessory protein. An exception to this may be the case of certain Golgi enzymes, with little to no substrate specificity, but with very high activity, like zDHHC3 (32). Indeed, enhancement of GCP16 S-acylation was also observed with zDHHC3 coexpression. In addition, coexpression of zDHHC9 or zDHHC3 also enhanced the stability of GCP16, suggesting that S-acylation prevents GCP16 degradation. It is possible that the hydrophobicity of α2′ and α3′ helices initiate membrane attachment of GCP16, bringing the protein into proximity with membrane-bound zDHHC9 (or other related zDHHC enzymes). Here, the hydrophobicity of the cysteines themselves might be important for initial membrane interaction, and indeed a similar proposal was also made for cysteine-string protein and SNAP25 (31, 34), which, like GCP16, are exclusively S-acylated and need to associate with membranes to undergo S-acylation. GCP16 that is weakly membrane-associated might be sensitive to ubiquitination, and subsequent S-acylation could protect the amino acid residues linked to rapid degradation by promoting a tighter or deeper membrane integration of the helices and surrounding residues. It is likely that there is also some protective effect on GCP16 that arises due to protein complex assembly with zDHHC9, and indeed interaction with zDHHC9 (and other partner zDHHC enzymes) may also contribute to the initial membrane targeting of GCP16. Notwithstanding this, the effects of zDHHC3 on GCP16 clearly show that S-acylation is a main factor contributing to GCP16 stability.

The stabilization of GCP16 by zDHHC9/zDHHC3 is similar to the reported stabilization of Golga7b by zDHHC5 (35). Indeed, it was shown that Golga7b was rapidly degraded when the cysteine residues were substituted, and this protein could only be detected when MG132 was added. In contrast, the cysteine mutants of GCP16 were readily detectable in our study. It is possible that the larger EGFP tag used in our study imparts an increased stability to GCP16. However, even if this was the case, the analysis of EGFP-GCP16 still revealed clear differences in protein stability, including for specific GCP16 mutants and for GCP16 coexpressed with zDHHC9 or zDHHC3, showing that EGFP-GCP16 can report on changes in protein stability. Indeed, although GCP16 and Golga7b are highly related and likely have similar modes of binding to different zDHHC enzymes, these accessory proteins do not have the same effects on zDHHC5, zDHHC8, zDHHC9, zDHHC14 and zDHHC18 (24, 25). It will therefore be important to undertake detailed structural and functional characterization of each of these accessory proteins to understand their regulatory effects on the zDHHC family.

Finally, the work reported here also highlighted the importance of proper zDHHC9-GCP16 engagement for the function of the proteins in dendrite growth. Mutations in the *ZDHHC9* gene cause intellectual disability and epilepsy in humans (18, 19), and it was shown that zDHHC9 knockdown led to reduced dendrite growth and inhibitory synapse formation (20), potentially explaining its functional role in brain development. In addition to effects on zDHHC9 knockdown, it was also shown that increasing the expression of both zDHHC9 and GCP16 (but not either protein alone) led to an increase in dendrite growth (20). The results of the present study extend these observations by showing that protein complex formation between coexpressed GCP16 and zDHHC9 is important for dendrite growth. The reduced functionality of the GCP16 interface mutant 3 is presumably linked to changes in the S-acylation of the proteins rather than their stability, as we selected neurons for analysis that had similar expression levels. In particular, the reduced stabilization of zDHHC9 S-acylation by this GCP16 mutant, and resulting effects on Ras S-acylation, is most likely to underlie the functional defects in dendrite growth. It is interesting to speculate on the timing of the formation of the zDHHC9-GCP16 complex. Polytopic membrane proteins such as zDHHC9 undergo cotranslational insertion initially in the ER membrane during their biosynthesis. Whether the GCP16-zDHHC9 complex forms at this early stage or only upon delivery of zDHHC9 to the Golgi has not been investigated. One interesting possibility is that limiting zDHHC9-GCP16 complex formation at the ER could restrict enzymatic activity as it moves through this compartment. Alternatively, zDHHC9 might be incompetent for ER exit until it has formed a stable complex with GCP16. In neurons specifically, it will be interesting to also explore the dynamics of GCP16-zDHHC9 complex formation on Golgi outposts in dendrites, where zDHHC9 regulates dendrite growth and synapse formation. Again, the timing of complex formation and regulation of zDHHC9 stability and activity might play a fundamental role in neuronal development.

## Experimental procedures

### Cell culture and transfection

HEK293T cells (ATCC, VA,) were cultured in Dulbecco's Modified Eagle Medium (DMEM)/GlutaMAX medium (Gibco, LifeTechnologies Ltd), supplemented with 10% fetal bovine serum (Gibco), at 37 °C and 5% CO_2_. Cells were plated on Corning poly-D-Lysine-coated 24-well plates (Thermo Fisher Scientific) for S-acylation, cycloheximide chase, and MG132 assays, or Corning poly-D-Lysine-coated 6-well plates (Thermo Fisher Scientific) for cell fractionations. Plasmid DNA was introduced into the cells by transfection with polyethylenimine (Alfa Aesar stock at 1 mg/ml), at a ratio of 2 μl per μg plasmid DNA. For cells on 24-well plates, 0.6 μg of plasmid encoding zDHHC enzyme (or empty plasmid) was cotransfected with 0.4 μg of plasmid encoding EGFP-GCP16 (or EGFP). For 6-well plates, 2.4 μg of plasmid encoding zDHHC enzyme was cotransfected with 1.6 μg of plasmid encoding EGFP-GCP16. Cells were incubated at 37 °C for approximately 24 h before experimental analysis.

zDHHC enzyme constructs cloned in the pEF-BOS-HA vector were provided by Professor Masaki Fukata (36). Human GCP16 and mutants were synthesized and cloned into either pcDNA3.1(+)-N-eGFP or pcDNA3.1(+)-N-3x FLAG by GenScript (GenScript Biotech Ltd).

### S-acylation assays

Approximately 24 h after transfection, HEK293T cells were incubated for 4 h in media containing 50 μM of either palmitic acid (Sigma-Aldrich) or palmitic acid azide (described in (37)). Cells were then washed in PBS and scraped from plates in buffer containing 50 mM Tris pH 8.0, 0.5% SDS, and protease inhibitor cocktail (Sigma-Aldrich), and added to 1.5 ml tubes. The lysate (100 μl) was then mixed with 80 μl of a solution containing 2 mM CuSO_4_, 0.2 mM TBTA, and 200 μM alkyne-mPEG (Sigma-Aldrich), followed by the addition of 20 μl 40 mM ascorbic acid. Samples were incubated at room temperature for 1 h with end-over-end rotation, before the addition of SDS dissociation buffer for analysis by SDS-PAGE and immunoblotting. For quantification, the sum of all S-acylated bands was expressed as a % of the total protein signal (nonacylated plus S-acylated). The data were then normalized, and a mean value (± SD) was calculated for each condition from the duplicate palmitic acid azide samples.

### Cycloheximide chase assays

Approximately 24 h post-transfection, the media was aspirated, and cells were either washed and lysed in SDS dissociation buffer (0-h samples) or incubated with media containing 50 μg/ml cycloheximide for 8 h. The cycloheximide-treated cells were then washed in PBS and lysed in SDS dissociation buffer. Samples were analyzed by SDS-PAGE and immunoblotting. For quantification, the immunoreactive signal in each sample was expressed relative to the corresponding total protein stain (TPS; REVERT 700 TPS kit, LI-COR), and a mean value was calculated for each condition from the duplicate samples in each experiment. Then the % protein remaining after 8 h was determined by expressing the 8-h signal relative to the 0-h signal. A mean value (± SD) was calculated for each condition.

### MG132 proteasomal inhibition

Approximately 8 h after transfection, HEK293T cells on 24-well plates were incubated with 10 μM MG132 or a similar volume of DMSO. Around 16 h later, the media was aspirated, and cells were washed in PBS and lysed. Samples were then resolved by SDS-PAGE and analyzed by immunoblotting. Protein expression was normalized to the TPS levels of each sample, and expression levels after 16 h of MG132 treatment were quantified relative to the corresponding DMSO control values. A mean value (± SD) was calculated for each condition.

### Cell fractionation

Transfected cells were washed in PBS, scraped into 1.5 ml tubes, and centrifuged at 500×*g* for 3 min at 4 °C. The cell pellet was washed in PBS, resuspended in 200 μl of cold lysis buffer A (150 mM NaCl, 50 mM Hepes, 1 M hexylene glycol, and 25 μg/ml digitonin, pH 7.4) containing protease inhibitor cocktail, and incubated at 4 °C for 10 min, with end-over-end rotation. Samples were then centrifuged at 2000×*g* for 10 min, at 4 °C, and the supernatant (cytosol fraction) was collected and mixed with SDS dissociation buffer. The remaining pellets were dissolved in 267 μl of SDS dissociation buffer (membrane fractions). All samples were then analyzed by SDS-PAGE and immunoblotting. For quantification, protein membrane association was calculated as a percentage of the sum of the corresponding intensity values of the cytosolic and membrane fractions in each sample and the data were then normalized and a mean value (± SD) was calculated for each condition.

### Immunoblotting and antibodies

Proteins were transferred to nitrocellulose, and a TPS was performed before the membrane was scanned using a LI-COR Odyssey 9120 IR Imager (LI-COR Biosciences Ltd). The stain was removed from the nitrocellulose following the manufacturer’s instructions (LI-COR), which was then incubated in 5% non-fat milk in PBS containing 0.1% Tween-20 for 45 min, followed by incubation with primary antibody for 2 h and secondary antibody for 1 h before scanning on the LI-COR Odyssey. Mouse GFP antibody was used at 1:4000 (Takara Bio CA, USA; #632381); rat HA antibody was used at 1:1000 (Roche Diagnostics, Burgess Hill, UK; #11867423001); mouse FLAG antibody was used at 1:2000 (GenScript Biotech Ltd, Oxford, UK; #A00187); IRDye secondary antibodies were from LI-COR and were used at 1:20,000 dilution.

### Neuronal analysis

All experimental procedures involving animals were approved by the University of British Columbia Animal Care Committee and conducted in accordance with the guidelines of the Canadian Council on Animal Care (CCAC). Cultured rat hippocampal neurons from E18 Sprague-Dawley pups growing on coverslips were transfected at DIV 9 with 1.5 μg of each of HA-zDHHC9, FLAG-GCP16, and Camk2a-EGFP plasmid constructs using Lipofectamine 2000. Cells were then fixed at DIV 14 in 4% paraformaldehyde, 50 mM Hepes, and 4% sucrose in PBS for 10 min, washed three times in PBS for 10 min, and permeabilised in 0.1% Triton X-100 in PBS for 10 min. Cells were washed in PBS and blocked in 10% goat serum (GS) in PBS for 1 h at room temperature, and then incubated with primary antibody (1:500; rabbit monoclonal anti-HA (C29F4; Cell Signalling Technologies #3724) and mouse monoclonal anti-FLAG (9A3; Cell Signalling Technologies #8146) in 1% GS in PBS overnight at 4 °C, in the dark. After washing in PBS, cells were incubated in secondary antibodies (1:500; goat anti-rabbit Alexa Fluor Plus 647 (A32733) and goat anti-mouse (H + L) Alexa Fluor 568 (A11031), both from Life Technologies) in 1% GS in PBS for 1 h at room temperature, in the dark. During this incubation step, DAPI (1:5000) was added to each coverslip for the final 30 min. Coverslips were then washed in PBS and mounted on microscope slides using a drop of ProLong Gold Antifade Mountant. Cells were imaged on an Evident IX83 inverted microscope equipped with a Hamamatsu Orca Flash sCMOS camera and a X-Cite white light LED. Dendritic morphology was visualized using single 16 bit snaps using a 20x/0.8 NA UPLANAPO air objective with the camera in a 23 MHz readout mode. All images were acquired with equal LED settings and exposure times. To measure total dendritic length, .vsi files of imaged neurons at 20X magnification were imported into Image J software (version v1.54p) (https://imagej.net/) using the Bio-Formats Importer. The EGFP channel was manually thresholded and binarized into a mask. The mask was then imported into the SimpleNeuriteTracer plugin (38), and dendritic arbors were manually traced. The total dendritic length was then measured in SimpleNeuriteTracer. The scales for the distance were automatically calibrated based on the metadata for each image. The mean total dendritic length (± SD) was then calculated for each condition. The mean gray value for the FLAG-GCP16 WT and mutant construct and HA-ZDHHC9 channels was also measured within the EGFP mask in ImageJ as an indication of protein intensity.

## Data availability

All data are contained within the manuscript and are available upon request by corresponding author Luke H Chamberlain, at luke.chamberlain@strath.ac.uk.

## Supporting information

This article contains supporting information (21).

## Conflict of interest

The authors declare that they have no conflicts of interest with the contents of this article.
