# Supplementary material for: The GCP16-zDHHC9 S-acyltransferase complex: Reciprocal effects on protein S-acylation and stability
Source: J Biol Chem. 2026 Jul 21;302(9):113349. doi: 10.1016/j.jbc.2026.113349 (PMC13499115; doi:10.1016/j.jbc.2026.113349)
Supplement: Supplementary file 1 [file mmc1.docx]

**The GCP16-zDHHC9 S-acyltransferase complex: bidirectional effects on protein S-acylation and stability**

Despoina Allagioti^1^, Andrew J. Thompson^2^, Mohammad A.A.M. Al-Mhadeen^3^, Kimon Lemonidis^4^, Christine Salaun^1^, Nicholas C.O. Tomkinson^5^, Rami N. Hannoush^6^, Shernaz X. Bamji^2^, Luke H. Chamberlain^1^

**Supporting Information**

Figure S1. Interacting residues within the binding interfaces of zDHHC9 and GCP16.

Figure S2. Analysis of GCP16 S-acylation by zDHHC5, zDHHC14 and zDHHC19.

Figure S3. GCP16 cysteine residues at positions 69 and 72 are important for GCP16 membrane association.

Figure S4. GCP16 truncation mutants 1-90 and 1-120 are not S-acylated and fail to stabilise zDHHC9 S-acylation.

Figure S5. Effects of amino acid substitutions in all binding interfaces of zDHHC9 on the S-acylation of zDHHC9 and GCP16.

Figure S6. Effects of amino acid substitutions in all binding interfaces of GCP16 on the S-acylation of GCP16 and zDHHC9.

Figure S7. Effects of amino acid substitutions in all binding interfaces of zDHHC9 on the stability of zDHHC9 and GCP16.

Figure S8. Effects of amino acid substitutions in all binding interfaces of GCP16 on the stability of GCP16 and zDHHC9.

**Figure S1. Interacting residues within the binding interfaces of zDHHC9 and GCP16. (A)** Schematic showing the positions of the four binding interfaces within GCP16 and zDHHC9 (*top*) and a table highlighting the interacting residues within each interface of the zDHHC9-GCP16 protein complex (*bottom*), as reported by the cryo-EM structure published by Yang *et al.* (2024). **(B)** Cartoon representation of the zDHHC9-GCP16 protein complex (PDB: 8HF3). zDHHC9 is shown in slate blue and GCP16 in salmon. The surface of zDHHC9 is shown in light grey. The interacting residues of the identified binding interfaces are depicted. Residues within GCP16 are annotated in black, and cysteine residues are highlighted in turquoise, while zDHHC9 residues are annotated in plum and cysteine residues are highlighted in yellow. The TM domains of zDHHC9 are highlighted in pink. Enlarged images show a close-up view of each interface numbered 1-4 and the residues involved, along with key residues in proximity. Hydrogen bonding is depicted using green dotted lines.

**Figure S2. Analysis of GCP16 S-acylation by zDHHC5, zDHHC14 and zDHHC19.** HEK293T cells were co-transfected with plasmids encoding EGFP-GCP16 together with either PEF-BOS-HA empty plasmid as a control or **(A)** HA-zDHHC5, **(B)** HA-zDHHC14, or **(C)** HA-zDHHC19. Cells were labelled with palmitic acid azide (Az-C16:0) (Az) or palmitic acid (C16:0) as a control (C) for 4 hours and were then lysed and clicked using Alkyne-mPEG. Samples were resolved by SDS-PAGE, transferred to nitrocellulose, and analysed by a total protein stain (TPS) followed by immunoblotting with GFP and HA antibodies. S-acylation is indicated by band shifts in Az samples. The position of molecular weight markers (kDa) is shown on the left of all immunoblots. Quantified data show normalised protein S-acylation (mean ± SD). S-acylation (%) was calculated as a proportion of acylated and non-acylated band intensities. Data were normalised to the highest value in each experiment (set to 1), then duplicates were averaged. Statistical significance was determined using a t-test. ***p<0.001, while ns denotes non-significance, where p>0.05 (p values in panels (A) and (B) were <0.0001, while p = 0.8329 in panel (C)). n = 4, from four independent experiments.

**Figure S3. GCP16 cysteine residues at positions 69 and 72 are important for GCP16 membrane association.** HEK293T cells were co-transfected with EGFP-GCP16 WT, GCP16 (C69A), GCP16 (C72A), or GCP16 (C69A/C72A), together with either **(A)** PEF-BOS-HA empty plasmid (control) or **(B)** HA-zDHHC9. Cells were fractionated to separate the cytosolic from membrane proteins, and fractions were resolved by SDS-PAGE, transferred to nitrocellulose, and analysed by a total protein stain (TPS) followed by immunoblotting with the appropriate antibodies. GAPDH was used as a cytosolic marker, while calnexin was used as a membrane marker. The position of molecular weight markers (kDa) is shown on the left of all immunoblots. Quantified data show normalised membrane association (mean ± SD). For each sample, the intensity of the membrane fraction was calculated as a percentage of the total signal (membrane + cytosolic). These values were then normalised within each experiment to the maximum value (set to 1). Statistical significance was determined using one-way ANOVA, followed by a Dunnett’s multiple comparisons test. **p<0.01, while ns denotes non-significance, where p>0.05 (p values in panel (A) were 0.9059 (C69A), 0.9193 (C72A), and 0.0021 (C69A/C72A), and in panel (B) were 0.3564 (C69A), 0.8954 (C72A), and 0.0027 (C69A/C72A)). n = 3, from two independent experiments.

**Figure S4. GCP16 truncation mutants 1-90 and 1-120 are not S-acylated and fail to stabilise zDHHC9 S-acylation.** HEK293T cells were co-transfected with EGFP-GCP16 WT, GCP16 (1-90), or GCP16 (1-120), together with **(A)** PEF-BOS-HA empty plasmid (control), or **(B)** HA-zDHHC9. Cells were labelled with palmitic acid azide (Az-C16:0) (Az) or palmitic acid (C16:0) as a control (C) for 4 hours and were then lysed and clicked using Alkyne-mPEG. Samples were resolved by SDS-PAGE, transferred to nitrocellulose, and analysed by a total protein stain (TPS) followed by immunoblotting with GFP and HA antibodies. S-acylation is indicated by band shifts in Az samples. The position of molecular weight markers (kDa) is shown on the left of all immunoblots. Quantified data show normalised protein S-acylation (mean ± SD). S-acylation (%) was calculated as a proportion of acylated and non-acylated band intensities. Data were normalised to the highest value in each experiment (set to 1), then duplicates were averaged. Statistical significance was determined using one-way ANOVA, followed by a Dunnett’s multiple comparisons test. ***p<0.001 (in panel (A), p values were <0.0001, while in panel (B) they were 0.0009 (1-90) and 0.0010 (1-120)). n = 3, from two independent experiments.

**Figure S5. Effects of amino acid substitutions in all binding interfaces of zDHHC9 on the S-acylation of zDHHC9 and GCP16. (A)** Schematic showing the positions of the four binding interfaces (*top*) and a table highlighting the amino acid changes introduced into the binding interfaces of zDHHC9 (*bottom*), as reported by the cryo-EM zDHHC9-GCP16 structure published by Yang *et al.* (2024). HEK293T cells were co-transfected with plasmids encoding **(B)** HA-zDHHC9 WT or HA-zDHHC9 1-4 binding interface mutant, together with EGFP or EGFP-GCP16, or with **(C)** EGFP-GCP16 together with PEF-BOS-HA empty plasmid as a control, HA-zDHHC9 WT, or HA-zDHHC9 1-4. Cells were labelled with palmitic acid azide (Az-C16:0) (Az) or palmitic acid (C16:0) as a control (C) for 4 hours and were then lysed and clicked using Alkyne-mPEG. Samples were resolved by SDS-PAGE, transferred to nitrocellulose, and analysed by a total protein stain (TPS) followed by immunoblotting with GFP and HA antibodies. S-acylation is indicated by band shifts in Az samples. The position of molecular weight markers (kDa) is shown on the left of all immunoblots. Quantified data show normalised protein S-acylation (mean ± SD). S-acylation (%) was calculated as a proportion of acylated and non-acylated band intensities. Data were normalised to the highest value in each experiment (set to 1), then duplicates were averaged. Statistical significance was determined using an unpaired t-test (panel B) or one-way ANOVA, followed by a Dunnett’s multiple comparisons test (panel C). *p<0.05 and ***p<0.001, while ns denotes non-significance, where p>0.05 (p values were 0.0233 (zDHHC9 WT) and 0.9764 (zDHHC9 Mut 1-4) in panel (B), and 0.0003 (zDHHC9 WT) and 0.2586 (zDHHC9 Mut 1-4) in panel (C)). n = 3, from three independent experiments.

**Figure S6. Effects of amino acid substitutions in all binding interfaces of GCP16 on the S-acylation of GCP16 and zDHHC9. (A)** Schematic showing the positions of the four binding interfaces and cysteine residues (*top*) and a table highlighting the amino acid changes introduced into the binding interfaces of GCP16 (*bottom*), as reported by the cryoEM zDHHC9-GCP16 structure published by Yang *et al.* (2024). **(B)** HEK293T cells were co-transfected with plasmids encoding HA-zDHHC9, together with EGFP, EGFP-GCP16 WT, or GCP16 1-4 binding interface mutant. Cells were labelled with palmitic acid azide (Az-C16:0) (Az) or palmitic acid (C16:0) as a control (C) for 4 hours and were then lysed and clicked using Alkyne-mPEG. Samples were resolved by SDS-PAGE, transferred to nitrocellulose, and analysed by a total protein stain (TPS) followed by immunoblotting with GFP and HA antibodies. S-acylation is indicated by band shifts in Az samples. The position of molecular weight markers (kDa) is shown on the left of all immunoblots. Quantified data show normalised protein S-acylation (mean ± SD). S-acylation (%) was calculated as a proportion of acylated and non-acylated band intensities. Data were normalised to the highest value in each experiment (set to 1), then duplicates were averaged. Statistical significance was determined using an unpaired t-test (top graph) or one-way ANOVA, followed by a Dunnett’s multiple comparisons test (bottom graph). *p<0.05 and ***p<0.001, while ns denotes non-significance, where p>0.05 (p values were 0.0003 (top graph), and 0.0101 (WT) and 0.1848 (Mut 1-4) in the bottom graph). n = 3, from three independent experiments.

**Figure S7. Effects of amino acid substitutions in all binding interfaces of zDHHC9 on the stability of zDHHC9 and GCP16.** HEK293T cells were co-transfected with plasmids encoding **(A)** HA-zDHHC9 WT or HA-zDHHC9 1-4 binding interface mutant, together with EGFP or EGFP-GCP16, or with **(B)** EGFP-GCP16, together with HA-zDHHC9 WT, or HA-zDHHC9 1-4. Cell lysates were collected at 0 hours or after 8 hours of incubation with 50 µg/ml cycloheximide (CHX). Samples were resolved by SDS-PAGE, transferred to nitrocellulose, and analysed by a total protein stain (TPS) followed by immunoblotting with GFP and HA antibodies. The position of molecular weight markers (kDa) is shown on the left of all immunoblots. Percentage protein expression after 8 hours of CHX treatment is shown for each condition (mean ± SD). Signals were normalised to the total protein stain (TPS) for each lane and then expressed relative to the corresponding 0h value (set to 100%). Statistical significance was determined using unpaired t-tests. **p<0.01, while ns denotes non-significance, where p>0.05 (p values in panel (A) were 0.0016 (zDHHC9 WT) and 0.6377 (zDHHC9 Mut 1-4), and in panel (B) the p value was 0.0088). n = 4, from three independent experiments.

**Figure S8. Effects of amino acid substitutions in all binding interfaces of GCP16 on the stability of GCP16 and zDHHC9. (A)** HEK293T cells were co-transfected with plasmids encoding HA-zDHHC9, together with EGFP, EGFP-GCP16 WT, or GCP16 1-4 binding interface mutant. Cell lysates were collected at 0 hours or after 8 hours of incubation with 50 µg/ml cycloheximide (CHX). Samples were resolved by SDS-PAGE, transferred to nitrocellulose, and analysed by a total protein stain (TPS) followed by immunoblotting with GFP and HA antibodies. The position of molecular weight markers (kDa) is shown on the left of all immunoblots. **(B-C)** Percentage protein expression after 8 hours of CHX treatment is shown for each condition (mean ± SD). Signals were normalised to the total protein stain (TPS) for each lane and then expressed relative to the corresponding 0h value (set to 100%). Statistical significance was determined using an unpaired t-test (B) or one-way ANOVA, followed by a Dunnett’s multiple comparisons test (C). ***p<0.001, while ns denotes non-significance, where p>0.05 (p values were 0.0002 in panel (B), and 0.0002 (WT), and 0.8206 (Mut 1-4) in panel (C)). n = 4, from four independent experiments.
